# The challenges of selective fertility and carryover effects in within-sibship analyses: the effect of assisted reproductive technology on perinatal mortality as an example

**DOI:** 10.1093/ije/dyad003

**Published:** 2023-01-28

**Authors:** Kjersti Westvik-Johari, Siri E Håberg, Deborah A Lawlor, Liv Bente Romundstad, Christina Bergh, Ulla-Britt Wennerholm, Mika Gissler, Anna-Karina A Henningsen, Aila Tiitinen, Anja Pinborg, Signe Opdahl

**Affiliations:** Department of Fertility, Women and Children’s Centre, St Olavs Hospital, Trondheim, Norway; Department of Public Health and Nursing, Norwegian University of Science and Technology, Trondheim, Norway; Centre for Fertility and Health, Norwegian Institute of Public Health, Oslo, Norway; Centre for Fertility and Health, Norwegian Institute of Public Health, Oslo, Norway; MRC Integrative Epidemiology Unit at the University of Bristol, Bristol, UK; Population Health Science, Bristol Medical School, Bristol, UK; NIHR Bristol Biomedical Research Centre, Bristol, UK; Centre for Fertility and Health, Norwegian Institute of Public Health, Oslo, Norway; Spiren Fertility Clinic, Trondheim, Norway; Department of Obstetrics and Gynaecology, Institute of Clinical Sciences, Sahlgrenska Academy, University of Gothenburg, Sahlgrenska University Hospital, Gothenburg, Sweden; Department of Obstetrics and Gynaecology, Institute of Clinical Sciences, Sahlgrenska Academy, University of Gothenburg, Sahlgrenska University Hospital, Gothenburg, Sweden; Department of Knowledge Brokers, THL Finnish Institute for Health and Welfare, Helsinki, Finland; Department of Molecular Medicine and Surgery, Karolinska Institutet, Stockholm, Sweden; Academic Primary Health Care Centre, Region Stockholm, Stockholm, Sweden; Fertility Clinic, Copenhagen University Hospital, Rigshospitalet, Copenhagen, Denmark; Department of Obstetrics and Gynaecology, Helsinki University Hospital and University of Helsinki, Helsinki, Finland; Fertility Clinic, Copenhagen University Hospital, Rigshospitalet, Copenhagen, Denmark; Department of Public Health and Nursing, Norwegian University of Science and Technology, Trondheim, Norway

**Keywords:** Cohort study, sibship design, assisted conception, perinatal death, perinatal mortality, selection bias, selective fertility, carryover effects, continuation rate

## Abstract

**Background:**

Within-sibship analyses show lower perinatal mortality after assisted reproductive technology (ART) compared with natural conception (NC), a finding that appears biologically unlikely. We investigated whether this may be attributed to bias from selective fertility and carryover effects.

**Methods:**

Using data from national registries in Denmark (1994–2014), Finland (1990–2014) and Norway and Sweden (1988–2015), we studied 5 722 826 singleton pregnancies, including 119 900 ART-conceived and 37 590 exposure-discordant sibships. Perinatal mortality at the population level and within sibships was compared using multilevel logistic regression with random and fixed intercepts, respectively. We estimated selective fertility as the proportion of primiparous women with and without perinatal loss who had a second delivery, and carryover effects through bidirectional and crosswise associations.

**Results:**

Population analysis showed higher perinatal mortality among ART conception compared with NC (odds ratio 1.21, 95% CI 1.13 to 1.30), whereas within-sibship analysis showed the opposite (OR 0.36, 95% CI 0.31 to 0.43). Primiparous women with perinatal loss were more likely to give birth again (selective fertility) and to use ART in this subsequent pregnancy (carryover effects), resulting in strong selection of double-discordant sibships with death of the naturally conceived and survival of the ART-conceived sibling. After controlling for conception method and outcome in the first pregnancy, ART was not consistently associated with perinatal mortality in the second pregnancy.

**Conclusions:**

Whereas population estimates may be biased by residual confounding, within-sibship estimates were biased by selective fertility and carryover effects. It remains unclear whether ART conception contributes to perinatal mortality.

Key Messages

- Previous studies show higher perinatal mortality in pregnancies after assisted reproductive technology (ART) compared with natural conceptions (NC) but may be biased by residual confounding.
- Within-sibship analyses are less prone to parental confounding, including factors related to subfertility, but in three previous studies perinatal mortality was lower for ART-conceived pregnancies in within-sibship analyses, and the authors discussed that these analyses could be biased.
- These findings were likely biased by selective fertility (increased likelihood of conceiving again after a pregnancy loss) and carryover effect (here increased likelihood of ART-conception following loss with NC), but the studies were unable to control for this.
- We found that women with perinatal loss were more likely to conceive again (selective fertility) and to conceive by ART in their second pregnancy, particularly when the firstborn was conceived naturally, compared with women with a surviving child (carryover effects).
- Our study is an example of how strong selection can bias within-sibship designs and that very large sample sizes are needed to control these biases.

## Introduction

Aetiological studies with observational data are prone to residual confounding,^1^ and within-sibship comparison is a valuable approach to reduce this source of bias through control for all measured and unmeasured factors that are shared by siblings, such as genetics and parental health, lifestyle and socioeconomic position.^2^ If results from conventional population analysis and within-sibship comparison are comparable, causal inference is strengthened, whereas differing results require consideration of other explanations and may indicate the presence of bias in either the conventional population or the sibling analyses.^3^ A within-sibship comparison is based on sibships with differences in exposure (e.g. one sibling is exposed and the other is not), often referred to as exposure-discordant sibships. For dichotomous outcomes, the comparison further requires that the outcome differ within each sibship (e.g. one sibling experiences the outcome and the other does not). The crucial role of sibships where both the exposure and the outcome differ, so-called double-discordant sibships, makes within-sibship comparisons of binary measures particularly vulnerable to bias from mechanisms that increase or reduce the prevalence of these sibships.^4^ Examples of such mechanisms include bias from misclassification, from confounding by factors that differ between siblings (i.e. non-shared confounding)^5^ and from siblings influencing each other’s exposure levels or outcomes, referred to as carryover effects or contagion.^6^ Non-shared confounding is less likely for early-life exposures than for exposures later in life.^7^ In contrast, carryover effects might be expected whenever exposures or outcomes for one sibling affect antenatal monitoring, obstetric management or diagnostic awareness for subsequent siblings.

Within-sibship comparisons have been applied to strengthen causal inference on child and maternal health outcomes of pregnancies conceived by assisted reproductive technology (ART) and to disentangle the contributions from parental subfertility and treatment factors.^8–11^ Conventional population analyses show that ART-conceived singletons have a higher risk of preterm birth and low and high birthweight compared with those naturally conceived, and these associations are supported by consistent results from within-sibship analyses, suggesting that confounding by parental factors is limited and that the associations may reflect causality. In contrast, the higher perinatal mortality seen in ART-conceived pregnancies at the population level^12^^,^^13^ has been accompanied by strong negative associations in three previous within-sibship comparisons.^9–11^ Given that ART appears to increase the risk of preterm birth and low birthweight, it seems biologically unrealistic that ART should prevent perinatal death. Of note, in all three previous studies, which included 2204,^9^ 3879^10^ and 1813^11^ discordant sibling pairs, the within-sibship results seemed to be driven primarily by higher perinatal mortality in natural conceptions that were followed by an ART-conceived pregnancy. One study also showed that among women with a first natural conception, the next pregnancy was more likely to be ART-conception if perinatal death occurred in the first pregnancy compared with when the firstborn survived.^9^ Although the results were not interpreted as causal in any of these previous studies, the potential mechanisms of bias were only briefly explored.^9^^,^^10^

It is possible that these results were biased by the combined effects of selective fertility and carryover. Selective fertility refers to the observed evidence that couples who experience perinatal death are more likely to have subsequent pregnancies than couples with a surviving child.^14^ It seems plausible that for some of these couples, the wish for a new pregnancy may lead to ART treatment, thereby introducing a carryover effect where the outcome of the first sibling influences the exposure in the subsequent sibling.^6^ These two selection forces would increase the occurrence of sibships that are discordant for both exposure and outcome. Proposed strategies to identify bias from carryover effects within sibships include bidirectional analysis (i.e. interaction with birth order) and testing for crosswise associations (e.g. association of outcome in first pregnancy with exposure in subsequent pregnancies).^6^^,^^15^ If present, these biases might be reduced by keeping the selection forces constant, for example by comparing perinatal mortality between ART and natural conception in the second pregnancy for women with the same conception method and outcome in their first pregnancy. In the absence of other biases, a causal effect of ART could then be expected to result in consistently higher perinatal mortality in the second pregnancy. However, these methods are likely to need much larger samples than those available in previous studies.

The aim of this study was to investigate whether within-sibship results of a lower perinatal mortality for ART compared with natural conception could be due to bias from selective fertility and carryover effects. We use data from the Committee of Nordic ART and Safety (CoNARTaS) cohort on 5 722 826 participants, including 37 590 sibships discordant for ART and natural conception.

## Methods

### Data sources and study population

The CoNARTaS cohort includes data on all births registered in the nationwide Medical Birth Registries (MBRs) in Denmark (1994–2014), Finland (1990–2014), Norway (1984–2015) and Sweden (1985–2015).^16^ Because of very few deliveries from ART-conceptions before 1988, we restricted the study period to 1988–2014/15. Data were linked to information from national ART registries using the unique national identity number assigned to residents in the Nordic countries.^17^^,^^18^ Exposure was conception after ART treatment, defined as any fertilization outside the body with subsequent embryo transfer of fresh or frozen embryos.^19^ Pregnancies without registration of ART treatment were considered as naturally conceived and included non-ART fertility treatments such as insemination and ovulation induction with natural fertilization. Outcome was perinatal death, defined as stillbirth and neonatal death 0–27 days after birth.

**Figure 1:**
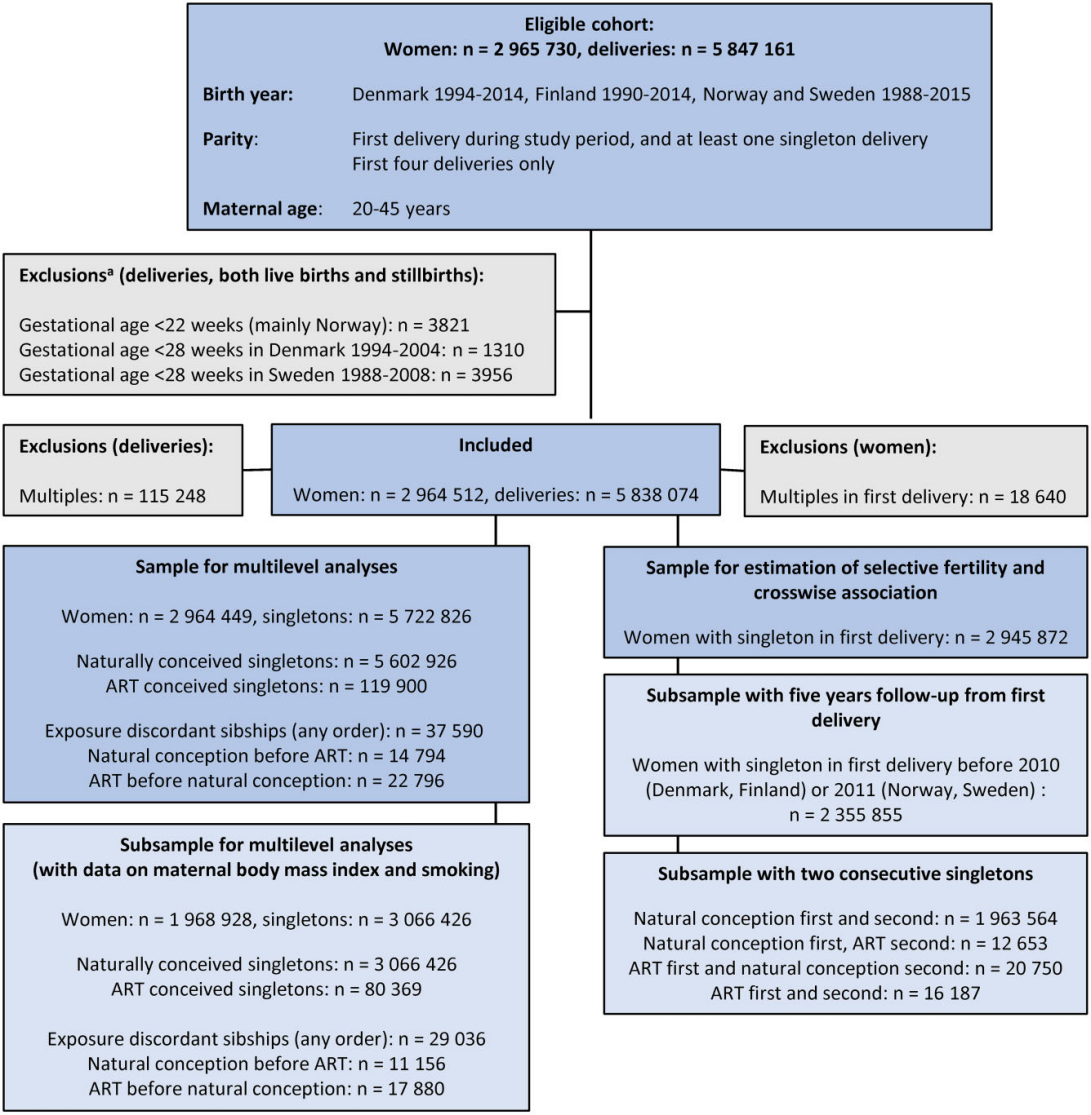
Overview of eligibility criteria, exclusion and inclusion in the study population. Registration of stillbirths differed over time and between the countries. In Sweden and Denmark, stillbirths were registered from ≥28 weeks until April 2004 in Denmark and July 2008 in Sweden, when the registration was changed to ≥22 weeks. In Finland and Norway, stillbirths ≥22 weeks were registered throughout the study period. Live births were registered at any gestational age throughout the study period in all countries. ART, assisted reproductive technology

Women who gave first birth during the study period, at ages 20–45 years, were eligible. For each woman, we included up to the first four deliveries. In population-level and within-sibship analyses, we included only singleton deliveries, and excluded deliveries at gestational ages below the definition thresholds for early and late fetal deaths^20^ in line with registration practices in each country, as described in Figure 1. This analysis sample comprised 5 722 826 singletons, including 119 900 ART-conceived singletons and 37 590 exposure-discordant sibships (i.e. sibships with at least one naturally conceived and at least one ART-conceived singleton, in any order). To estimate selective fertility and carryover effects, we included 2 945 872 women whose first delivery was a singleton.

### Statistical analysis

We first estimated perinatal mortality for ART vs natural conception at the population level and within sibships, using multilevel logistic regression with pregnancies as one level and mothers as another (i.e. siblings nested within mothers).^21^ Population-level associations were estimated in random intercept models using the full analysis sample, where each woman contributed 1–4 deliveries. Within-sibship associations were estimated in models with a fixed intercept for each woman (also referred to as a ‘conditional’ model),^21^ using discordant sibships with each woman contributing 2–4 deliveries. We estimated odds ratios (ORs) with 95% confidence intervals (CIs) and calculated risk differences (RDs) from predicted risks. Potential confounders were factors which could influence perinatal mortality and the need for ART treatment. We included the following covariates: year of birth, country, maternal age and parity. For pregnancies with available information, we also adjusted for maternal height, pre-pregnancy or first trimester body mass index (BMI, kg/m^2^) and any smoking during pregnancy in a sub-population. Within-sibship estimates were not adjusted for constant factors (maternal height and country). In sensitivity analyses, we also investigated the association among full siblings (same mother and father) and separately for each country.

Next, we investigated if the results could be biased by selective fertility and carryover effects. A ‘bidirectional analysis’, where effect measure modification by order of exposure within sibships is examined, has been suggested as a method to identify some types of carryover effects, including the type where the outcome for the first sibling influences exposure in subsequent sibling(s).^6^ As a first step to investigate whether carryover effects were present in our analyses, we compared perinatal mortality in the mothers’ first two (consecutive) singleton deliveries for women with natural conception followed by ART (NC-ART) and women with ART followed by natural conception (ART-NC). From previous within-sibship studies,^9–11^ we would expect a carryover effect to result in a stronger association for the NC-ART group compared with ART-NC. For comparison, we also included estimates for women with only natural conception (NC-NC) or only ART-conception (ART-ART) in both pregnancies. Risk estimates for the resulting eight groups were obtained from random intercept models and with interaction terms between a variable containing birth order (first or second) and a variable containing the four combinations of conception method (NC-NC, NC-ART, ART-NC or ART-ART).

To estimate selective fertility,^14^ we calculated the proportion of women with a firstborn singleton who had a second delivery from either conception method, according to conception method and perinatal death in the first pregnancy. The proportions that had a second delivery were calculated over the full study period and within 5 years after first delivery, to account for the fact that more deliveries from ART-conception took place during the later years of the study period. Because a large proportion of ART-conceived pregnancies are multiples, we report the proportions who continued with a singleton or with a multiple delivery, to give a more complete overview of the selection into the within-sibship models. We expect bias from selective fertility to be present if continuation to a second delivery is higher after perinatal death than for those with a surviving singleton. To examine crosswise associations in the presence of selective fertility, we compared the probabilities of a second singleton delivery with each conception method for women with perinatal death or survival in the first pregnancy. If these probabilities differ, e.g. if women with a perinatal death have a higher probability of a second delivery from ART than women with a surviving child, this will indicate that carryover effects are present.

Finally, to control bias from selective fertility and carryover effects, we compared perinatal mortality for ART vs natural conception in the second singleton pregnancy for women who had the same conception method and outcome in the first pregnancy. We assume that among women who had the same experience in their first pregnancy (as far as the available data could indicate), the proportion wanting a subsequent pregnancy would be comparable, thereby limiting bias from selective fertility. We also assume that a potential contribution from ART-conception to perinatal mortality in the second pregnancy would be independent of the specific combinations of exposure and outcome in the first pregnancy, thereby limiting bias from carryover effects. In other words, if ART-conception increases perinatal mortality, we would expect that ART-conceived second pregnancies would have a higher perinatal mortality than naturally conceived second pregnancies, when holding the events of the first pregnancy constant. Although this approach takes advantage of repeated observations (pregnancies) for each woman, it should not be considered a sibship comparison, because the comparison is made between unrelated singletons. Instead, it may be helpful to consider this comparison a matched cohort study, where matching is performed on reproductive history.

## Results

Women who conceived by ART were older than those who conceived naturally, regardless of pregnancy outcome (Table 1). For both conception methods, pregnancies resulting in perinatal death had higher mean maternal BMI, and maternal smoking and preterm birth (<37 weeks) was more common, than in pregnancies with a surviving singleton (Table 1;Supplementary Table S1, available as Supplementary data at *IJE* online).

**Table 1:** Maternal characteristics of the full sample for multilevel analyses, according to conception method and perinatal survival status

		Natural conception		Assisted reproductive technology	
		Perinatal survival	Perinatal death	Perinatal survival	Perinatal death
Singleton deliveries, total	*n* (%)	5 573 672 (99.5)	29 254 (0.52)	119 090 (99.3)	810 (0.68)
Denmark		991 211 (99.4)	5742 (0.58)	28 471 (99.2)	237 (0.83)
Finland		1 103 523 (99.5)	5286 (0.48)	20 528 (99.3)	140 (0.68)
Norway		1 234 752 (99.4)	7748 (0.62)	22 280 (99.1)	203 (0.90)
Sweden		2 244 186 (99.5)	10 478 (0.46)	47 803 (99.5)	230 (0.48)
Maternal age, years	Mean (SD)	29.6 (4.8)	29.7 (5.1)	33.8 (4.2)	33.8 (4.3)
Primiparous	*n* (%)	2 843 092 (51.0)	16 621 (56.8)	85 515 (71.8)	644 (79.5)
Maternal body mass index, kg/m^2^^a^	Mean (SD)	24.2 (4.5)	25.4 (5.4)	24.2 (4.1)	25.3 (4.4)
	Missing (%)	2 455 204 (44.1)	16 129 (55.1)	37 722 (31.7)	385 (47.5)
Maternal smoking, yes^b^^,^^c^	*n* (%)	601 304 (12.5)	3829 (17.3)	5919 (5.4)	53 (7.9)
	Missing (%)	756 162 (13.6)	7170 (24.5)	9327 (7.8)	136 (16.8)

a Maternal height and weight before pregnancy or during first trimester were reported during the period 1988–89 and 1992–2015 in Sweden, 2004–14 in Denmark and Finland and 2007–15 in Norway, with substantial missing data during the first years of registration in all countries.

b Maternal smoking was registered throughout the study period in Denmark, Finland and Sweden and, since 1999, in Norway and was categorized as smoking or non-smoking.

c Percentages of the characteristic are calculated among those with available information, whereas percentages of missingness are calculated among the total number of observations.

At the population level, perinatal mortality was higher after ART compared with natural conception when adjusting for available confounders (OR 1.26, 95% CI 1.13 to 1.30, Table 2). Within sibships, we found a markedly lower perinatal mortality for ART-conceived singletons compared with their naturally conceived siblings (adjusted OR 0.36, 95% CI 0.31 to 0.43). Additional adjustment for maternal BMI and smoking, and restriction to full siblings, had little influence on associations. Reversal of the association from population level to within sibships was consistent across countries (Supplementary Table S2, available as Supplementary data at *IJE* online).

**Table 2:** Risk of perinatal death by conception method: population-level and within-sibship estimates for up to four singleton deliveries per woman

	Population estimates (random effects)						Within-sibship estimates (fixed effects)			
	Numbers	Risk^a^ %	RD^a^ pp	Adj. RD (95% CI)	OR^a^ (95% CI)	Adj. OR (95% CI)	Numbers^b^	Risk^a^ %	OR^a^	Adj. OR (95% CI)
**Main population**										
Natural	5 602 926	0.52	0	Ref.^c^	1	Ref.^c^	45 875	1.29	1	Ref.^d^
ART	119 900	0.68	0.15 (0.10–0.20)	0.11 (0.06 to 0.15)	1.30 (1.21–1.30)	1.21 (1.13 to 1.39)	40 085	0.72	0.57 (0.49 to 0.66)	0.36 (0.31 to 0.43)
**Sample with data on BMI and smoking**										
Natural	3 066 426	0.41	0	Ref.^e^	1	Ref.^e^	23 091	1.13	1	Ref.^f^
ART	80 369	0.51	0.1 (0.04 to 0.15)	0.04 (−0.01 to 0.09)	1.24 (1.12 to 1.37)	1.10 (0.99 to 1.22)	21 107	0.54	0.48 (0.38 to 0.60)	0.28 (0.22 to 0.37)
**Full siblings** ^g^ ^,^ ^h^										
Natural	3 471 670	0.23	0	Ref^c^	1	Ref^c^	32 205	0.49	1	Ref^d^
ART	53 571	0.30	0.06 (0.02–0.11)	0.09 (0.04 to 0.14)	1.28 (1.09–1.50)	1.41 (1.20 to 1.66)	28 252	0.28	0.55 (0.41 to 0.75)	0.32 (0.23 to 0.44)

Adj., adjusted; ART, assisted reproductive technology; BMI, body mass index; OR, odds ratio; pp, percentage points; RD, risk difference, Ref., reference.

a Unadjusted.

b Numbers refer to children who are part of a maternal sibling group with at least two different conception methods within the group.

c Adjusted for maternal age, parity, country, year of birth.

d Adjusted for maternal age, parity, and year of birth.

e Additionally adjusted for maternal BMI, height and smoking.

f Additionally adjusted for maternal BMI and smoking.

g Defined as siblings with the same mother and father; analyses are therefore restricted to couples with 2–4 children for both random and fixed effects.

h Data from Finland are not included because paternal identity indicator was not available.

**Figure 2:**
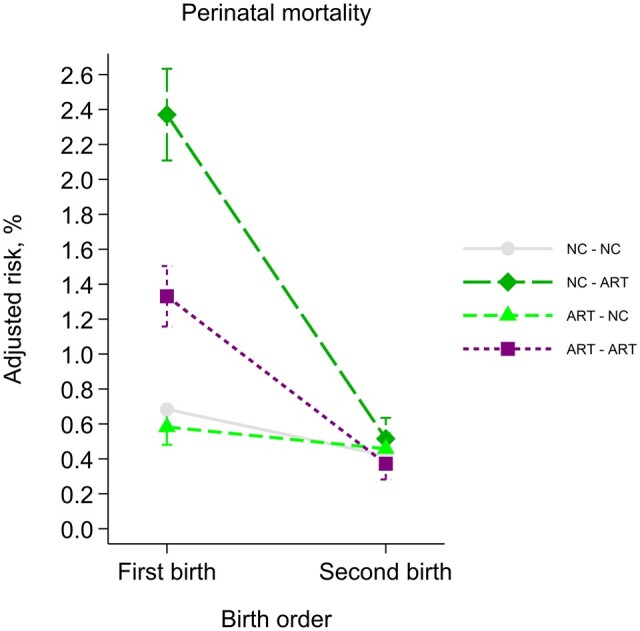
Perinatal death in first and second consecutive singleton delivery, according to conception method. Error bars represent 95% confidence intervals. NC, naturally conceived; ART, assisted reproductive technology

The naturally conceived singletons in sibships that were discordant on conception method, had higher perinatal mortality (1.29%) than naturally conceived at the population level (0.52%), whereas perinatal mortality was comparable between singletons born after ART-conception in both samples (0.72% in sibships vs 0.68% in the full population). Bidirectional analysis showed that this finding, as well as the within-sibship association, were driven mainly by women with natural conception before ART-conception (Figure 2), who had the highest perinatal mortality in first delivery and the steepest decline in perinatal mortality from first to second delivery. This heterogeneity suggests that a carryover effect may be present, but cannot directly identify the mechanism (e.g. if outcome in the first pregnancy influences exposure in the second).

Estimates of selective fertility showed that women with a perinatal loss in their first pregnancy were more likely to give birth again than women with a child who survived the perinatal period (Table 3). Specifically, for women with a naturally conceived first pregnancy, 70% of those with a surviving child proceeded with a second delivery, compared with 82.4% of women who experienced a perinatal loss. When the first pregnancy was conceived by ART, the corresponding percentages were 46.4% and 63.7%, respectively. Women who lost their naturally conceived firstborn were more than four times more likely to continue with an ART-conceived singleton pregnancy than women with a naturally conceived firstborn surviving child (1.8% vs 0.43%), indicating a strong carryover effect from the outcome in the first pregnancy to exposure in the next. Similarly, women with ART-conception in the first pregnancy were also more likely to continue with an ART-conceived singleton if the firstborn died (35.1% vs 18.7%). This suggests that carryover effects are also present when the firstborn is ART-conceived, resulting in selection of sibships where a surviving ART-conceived singleton is followed by natural conception. Patterns of selective fertility were similar when follow-up was restricted to 5 years (Supplementary Table S3, available as Supplementary data at *IJE* online).

**Table 3:** Continuation to a second delivery and risk of perinatal death in the second singleton pregnancy among women with a firstborn singleton, according to conception method and pregnancy outcome of the first delivery

Conception method and outcome in first, singleton delivery		Continuation to a second delivery, by conception method and plurality in second delivery			Perinatal mortality in second delivery	
			Full study period, *n* (%)		Risk, %	OR (95% CI)^a^
NC surviving child	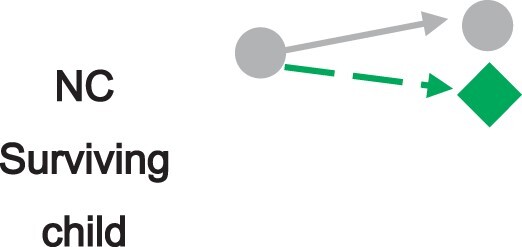	NC singleton	1 950 444	68.6	0.42	1 (ref)
		ART singleton	12 347	0.43	0.53	1.26 (0.99 to 1.61)
		NC multiples	25 655	0.90		
		ART multiples	2033	0.07		
		No continuation	852 613	30.0		
		Total	2 843 092	100		
NC perinatal loss	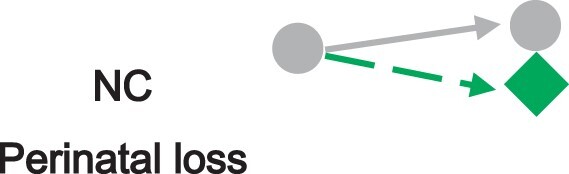	NC singleton	13 120	78.9	1.45	1 (ref)
		ART singleton	306	1.8	2.29	2.03 (0.93 to 4.45)
		NC multiples	228	1.4		
		ART multiples	47	0.28		
		No continuation	2920	17.6		
		Total	16 621	100		
ART surviving child	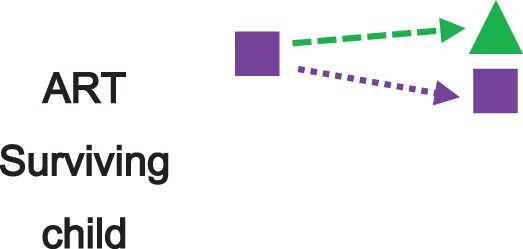	NC singleton	20 625	24.1	0.48	1 (ref)
		ART singleton	15 961	18.7	0.41	0.85 (0.62 to 1.17)
		NC multiples	652	0.76		
		ART multiples	2454	2.9		
		No continuation	45 823	53.6		
		Total	85 515	100		
ART perinatal loss	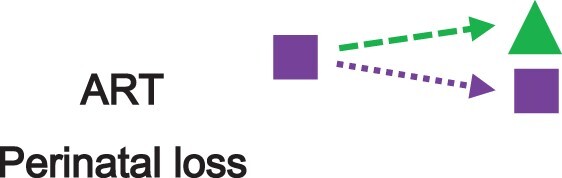	NC singleton	125	19.4	3.2	1 (ref)
		ART singleton	226	35.1	− ^b^	<1
		NC multiples	9	1.4		
		ART multiples	50	7.8		
		No continuation	234	36.3		
		Total	644	100		

Arrows and colours refer to the legend of Figure 2, to help illustrate how the selection into the discordant sibships occur.

ART, assisted reproductive technology; NC, natural conception; OR, odds ratio; ref, reference.

a Adjusted for maternal age, parity, year of birth and country.

b For data protection purposes, crude risk and estimates cannot be presented when based on a very low number of events.

To understand the impact of these selection mechanisms on the within-sibship association, we estimated perinatal mortality for the second-born singleton, according to conception method and outcome of the firstborn. Although the estimates were imprecise, they indicated no consistent pattern across the four groups: for ART vs NC in second pregnancy, perinatal mortality was higher when the first pregnancy was naturally conceived, and lower when the first pregnancy was ART-conceived (Table 3).

Further characterization of women with two consecutive singletons showed that women with NC-ART had longer time to first pregnancy than women with NC-NC, both with (mean 3.3 vs 2.6 years) and without (mean 2.8 vs 2.3 years) perinatal loss in first pregnancy (Supplementary Table S4, available as Supplementary data at *IJE* online). They also had the longest time between deliveries. For all four combinations of conception methods, time between deliveries was shorter, and interventions in second delivery were more frequent, when the firstborn died than when the firstborn survived the perinatal period.

## Discussion

### Summary of findings

We found that singletons conceived by ART had higher perinatal mortality than naturally conceived singletons at the population level. However, within sibships, perinatal mortality was much lower for ART-conceived compared with their naturally conceived siblings. Whereas the population analyses were potentially biased by residual confounding from parental factors, within-sibship analyses were biased because women with a perinatal loss were more likely to give birth a second time and were also more likely to conceive by ART in their second pregnancy than women without a perinatal loss. When accounting for conception method and outcome of the first pregnancy to control these biases, ART-conception was not consistently associated with perinatal mortality in the second pregnancy, but precision was low.

### Comparisons with other studies

Our population-level findings of higher perinatal mortality after ART compared with after NC are consistent with previous studies, including a meta-analysis from 2016 which comprised 106 267 ART-conceived and 1 262 997 NC-pregnancies (OR 1.64, 95% CI 1.41 to 1.90)^13^ and two more recent, but smaller studies.^22^^,^^23^ Using a discordant sibship cohort that is almost 10 times larger than previous studies, we confirm the results from within-sibship studies indicating lower perinatal mortality after ART compared with naturally conceived siblings.^9–11^ We also confirm their observations that the opposing results from population and within-sibship analyses may be attributed to a substantially higher mortality in naturally conceived pregnancies preceding an ART-conceived pregnancy. Romundstad *et al*. found that mothers who experienced a perinatal loss after natural conception were three times more likely to conceive by ART in the next pregnancy,^9^ and expressed caution in the interpretation of within-sibship estimates. Our findings additionally demonstrate that perinatal death increased the probability of another pregnancy, and that this pregnancy is more likely to be ART-conceived, regardless of conception method in the first pregnancy. Furthermore, conception method in the second pregnancy was not consistently associated with perinatal mortality when the history from the first pregnancy was accounted for.

### Strengths and limitations

In this large registry-based study from the Nordic countries, we investigated perinatal mortality among the ART-conceived compared with the naturally conceived, using both conventional and sibship designs and including a large sub-population with data on maternal BMI and smoking. The combination of selective fertility and carryover effects has not previously been explored as a source of bias in within-sibship analyses of outcomes in ART-conceived pregnancies. We were able to clearly demonstrate the presence of these biases, but despite the very large sample size, had insufficient power to estimate a precise effect of ART while controlling for the combined effect of selective fertility and carryover.

There are indications that sub- or infertility, even without fertility treatment, is associated with higher perinatal mortality.^24–26^ A subsample of data from Sweden indicated that the number of years trying to conceive was higher in first pregnancies with a perinatal loss compared with those without a perinatal loss, but data on an underlying infertility diagnosis were too limited to investigate the role of infertility further. The natural conception group included a small proportion of women with non-ART fertility treatment. If associations of ART with perinatal mortality in either the population or within-sibship analyses were driven by sub-fertility or non-ART fertility treatment, then these results might be biased towards the null, but the selective fertility and carryover effects would still increase the number of double-discordant sibships. It is also worth noting that if the underlying sub- or infertility was the main explanation of our results, we would expect perinatal mortality to be highest among women with ART-conception in their first pregnancy. Unfortunately, data on ART treatments not resulting in pregnancy, and on pregnancies from either conception method resulting in early miscarriages or terminations, were not available. We expect parents with previous perinatal loss to receive more intensive antenatal care, which could influence findings, for example through increased detection of fatal anomalies or higher probability of interventions, such as elective caesarean section to prevent intrauterine death. We did not have information to assess this.

Despite different definitions of stillbirth, the results from conventional and sibship comparisons were consistent between the countries. Similarities across the Nordic countries in population health and lifestyle, including pregnancy health,^27^ the public health care systems, the structure of registries and data linkage methods,^16^^,^^28^ add further support for pooling of data. Information on socioeconomic position and ethnicity, potentials confounders in the population analysis, was unfortunately not available but would be controlled for in the within-sibship analyses. Finally, we cannot exclude residual confounding from unmeasured, non-shared confounders, which may create stronger bias in within-sibship than in population-level analyses.^5^

### Implications of findings

Our study provides an example of strong selection bias in sibship comparisons. The strong selection into the double-discordant sibship group was driven by a combination of selective fertility, where women who experienced perinatal loss were more likely to have a subsequent delivery, and carryover effects, where perinatal death in one sibling increased the probability that the next sibling was ART-conceived.

These findings emphasize the importance of comparing population-level and within-sibship estimates and, if possible comparing these with other methods, to establish whether findings triangulate; where there are inconsistencies (as found here), it is essential to carefully consider biases in all approaches and not assume that conventional population analyses are most likely to be biased.^3^ In addition to the biases described here, sibship designs are prone to bias from misclassification and unmeasured, non-shared confounders.^4^ Furthermore, the plausibility of inconsistent results should be considered in relation to other evidence. We have previously shown that for other perinatal outcomes, namely preterm birth and small and large for gestational age, population and within-sibship estimates are consistent and support a causal effect of ART treatments.^8^ A possible explanation for the apparent difference in utility of within-sibship analyses between that study and the present study, is that perinatal loss is a dramatic and life-changing event for couples^29^ and an extreme outcome compared with other adverse perinatal outcomes. The probability of having only one birth is higher when the child survives despite other adverse perinatal outcomes, compared with when the child does not survive,^30^ suggesting that non-fatal adverse outcomes may not result in similarly strong selection.

In a broader context, selective fertility might be suspected in situations where a severe outcome in the first pregnancy, such as occurrence of congenital anomalies or severe maternal morbidity, will be known before the decision to pursue another pregnancy is made. Awareness of previous adverse outcomes may motivate lifestyle changes between pregnancies, or may prompt intensive clinical management in subsequent pregnancies,^31^ thereby creating opportunities for carryover effects between siblings.

Our alternative sibling approach, comparing perinatal mortality in the second pregnancy for women with the same experience in the first pregnancy, indicated opposite but very imprecise associations for women with NC and ART-conception in first pregnancy. The longer time to conception and to next delivery for women with perinatal loss in their first naturally conceived pregnancy, suggest that they did not seek ART for convenience, but due to infertility. Whether infertility contributed to the initial perinatal loss is unclear, but several gynaecological conditions are associated with preterm birth and other adverse perinatal outcomes, as well as with infertility.^32–35^ Moreover, causes of infertility may vary between couples with natural conception before ART (secondary infertility) and couples with ART-conception in their first pregnancy. To further advance our understanding of how ART treatment influences perinatal mortality, studies including even larger populations and information on causes of infertility may be helpful. From the current use of ART treatment in the Nordic countries, we expect that expanding the study period by another 5 years would increase the number of discordant sibships by about 30%. Taking advantage of the opportunities for family linkages in Nordic registries,^28^ comparison of perinatal mortality between sisters who conceived by different methods may overcome bias from selective fertility and carryover effects. This approach would assume that one sister’s perinatal loss will not affect decisions on parenthood or use of ART treatment for the other, while controlling for some family-level confounding.

## Conclusion

For the question of whether ART-conception influences perinatal mortality, conventional population-level and within-sibship analyses gave opposite results. Whereas conventional population level analyses may be biased by residual confounding, within-sibship analysis were biased by selective fertility and carryover effects. Alternative approaches to address this question further require even larger study populations.

## Ethics approval

In Denmark and Finland, ethical approval is not required for scientific projects solely based on registry data. In Norway, ethical approval was given by the Regional Committee for Medical and Health Research Ethics (REK-Nord, 2010/1909–1-24, 14398). In Sweden approval was obtained from the Ethical Committee in Gothenburg, Dnr 214–12, T422-12, T516-15, T233-16, T300-17, T1144-17 and T121-18.

## Supplementary Material

dyad003_Supplementary_Data

## Data Availability

The data underlying this article cannot be shared publicly due to the data protection and privacy of individuals who participated in the study. The procedures for data access are described in reference^16^ (Opdahl S, Henningsen AA, Bergh C *et al*. Data resource profile: the Committee of Nordic Assisted Reproductive Technology and Safety (CoNARTaS) cohort. *Int J Epidemiol* 2020; **49**:365–66f.).
