# Supplementary material for: The challenges of selective fertility and carryover effects in within-sibship analyses: the effect of assisted reproductive technology on perinatal mortality as an example
Source: Int J Epidemiol. 2023 Jan 28;52(2):403–13. doi: 10.1093/ije/dyad003 (PMC10266759; doi:10.1093/ije/dyad003)
Supplement: dyad003_Supplementary_Data [file dyad003_supplementary_data.docx]

| **Table S1. Pregnancy and treatment characteristics of the full sample for multilevel analyses, according to conception method and perinatal survival status** | | | | | |
| --- | --- | --- | --- | --- | --- |
|  | | **Natural conception** | | **Assisted reproductive technology** | |
|  |  | **Perinatal survival** | **Perinatal death** | **Perinatal survival** | **Perinatal death** |
| **Preterm birth <37 weeks ^a,b, c^** | n (%) | 254 708 (4.6) | 15 569 (56.2) | 8859 (7.4) | 517 (65.4) |
|  | missing (%) | 56 000 (1.0) | 1564 (5.4) | 242 (0.2) | 20 (2.5) |
| **Small for gestational age ^a, c^** | n (%) | 195 896 (3.6) | 7742 (32.1) | 5541 (4.7) | 201 (31.9) |
|  | missing (%) | 64 422 (1.2) | 5164 (17.7) | 432 (0.4) | 179 (22.1) |
| **Large for gestational age ^a, c^** | n (%) | 246 069 (4.5) | 1229 (5.1) | 4964 (4.2) | 30 (4.8) |
|  | missing (%) | 64 422 (1.2) | 5164 (17.7) | 432 (0.4) | 179 (22.1) |
| **Sex, girls** | n (%) | 2 712 424 (48.7) | 13 036 (46.4) | 58 201 (48.9) | 375 (48.8) |
| **Single embryo transfer ^a^** | n (%) | - | **-** | 48 650 (40.9) | 266 (32.8) |
|  | missing (%) | - | **-** | 33 912 (28.5) | 239 (29.5) |
| **Blastocyst transfer ^a^** | n (%) | - | **-** | 8180 (10.0) | 46 (8.9) |
|  | missing (%) | - | **-** | 37 506 (31.5) | 295 (36.4) |
| **^a^** Percentages of the characteristic are calculated among those with available information, whereas percentages of missingness are calculated among the total number of observations.  ^b^ For natural conceptions, gestational age was estimated from first (Denmark) or second (Norway and Sweden) trimester ultrasound examination when available, otherwise last menstrual period was used. For ART pregnancies, gestational age was estimated from transfer date in Sweden. In Denmark and Norway, gestational age was estimated from ultrasound examination, or transfer date if ultrasound data were missing.  ^c^ We used Marsal’s equations for intrauterine growth to estimate z-scores of birthweights where one standard deviation (SD) was set to 11% of the expected birthweight according to sex and gestational age in days, and small for gestational age was defined as -2 SD and large for gestational age was defined as +2 SD. | | | | | |

| **Table S2. Risk of perinatal death by conception method: population estimates and within sibship estimates, separately for each country.** | | | | | | | | | | | |
| --- | --- | --- | --- | --- | --- | --- | --- | --- | --- | --- | --- |
|  | **Population estimates (Random effects)** | | | | | |  | **Within sibship estimates (Fixed effects)** | | | |
|  | **Numbers** | **Risk ^a^, %** | **RD ^a^, *pp*** | **Adj. RD ^b^ (95% CI)** | **OR ^a^** (95%CI) | **Adj. OR ^b^ (95% CI)** |  | **Numbers ^c^** | **Risk ^a^, %** | **OR ^a^** | **Adj. OR ^b^ (95% CI)** |
| **Denmark** | | | |  |  |  |  |  |  |  |  |
| Natural | 996 953 | 0.58 | 0 | Ref | 1 | Ref |  | 9529 | 1.76 | 1 | Ref |
| ART | 28 716 | 0.83 | 0.24 (0.13 to 0.35) | 0.20 (0.10 to 0.30) | 1.44 (1.26 to 1.64) | 1.36 (1.18 to 1.56) |  | 8503 | 0.91 | 0.50 (0.38 to 0.67) | 0.35 (0.25 to 0.48) |
|  |  |  |  |  |  |  |  |  |  |  |  |
| **Finland** |  |  |  |  |  |  |  |  |  |  |  |
| Natural | 1 108 809 | 0.48 | 0 | Ref | 1 | Ref |  | 9969 | 1.04 | 1 | Ref |
| ART | 20 668 | 0.68 | 0.20 (0.08 to 0.31) | 0.16 (0.05 to 0.26) | 1.42 (1.20 to 1.69) | 1.33 (1.12 to 1.59) |  | 8263 | 0.81 | 0.85 (0.61 to 1.18) | 0.65 (0.46 to 0.92) |
|  |  |  |  |  |  |  |  |  |  |  |  |
| **Norway** |  |  |  |  |  |  |  |  |  |  |  |
| Natural | 1 242 500 | 0.62 | 0 | Ref | 1 | Ref |  | 8802 | 1.48 | 1 | Ref |
| ART | 22 483 | 0.90 | 0.27 (0.15 to 0.40) | 0.29 (0.16 to 0.41) | 1.46 (1.26 to 1.68) | 1.48 (1.27 to 1.71) |  | 7763 | 0.89 | 0.62 (0.45 to 0.84) | 0.34 (0.24 to 0.48) |
|  |  |  |  |  |  |  |  |  |  |  |  |
| **Sweden** |  |  |  |  |  |  |  |  |  |  |  |
| Natural | 2 254 664 | 0.46 | 0 | Ref | 1 | Ref |  | 17 576 | 1.07 | 1 | Ref |
| ART | 48 033 | 0.48 | 0.01 (-0.05 to 0.08) | -0.04 (-0.10 to 0.02) | 1.03 (0.90 to 1.17) | 0.91 (0.80 to 1.04) |  | 15 556 | 0.49 | 0.46 (0.35 to 0.60) | 0.27 (0.20 to 0.37) |
| Abbreviations: Adj. – adjusted, ART – assisted reproductive technology, BMI – body mass index, CI – confidence interval, OR – odds ratio, pp – percentage points, RD – risk difference, Ref. – reference.  ^a^ Unadjusted. ^b^ Adjusted for maternal age, parity, year of birth. ^c^ Numbers refer to children that are part of a maternal sibling group with at least two different conceptions methods within the group. | | | | | | | | | | | |

| **Table S3. Continuation to a second delivery among women with a firstborn singleton, according to conception method and pregnancy outcome of the first delivery. Follow-up restricted to 5 years.** | | | | |
| --- | --- | --- | --- | --- |
| **Conception method and outcome in first singleton delivery** | | **Continuation to a second delivery,**  **by conception method and plurality in second delivery** | | |
|  |  |  | **5 years follow-up, n (%)** | |
| **NC****Surviving child** |  | NC singleton | 1 544 783 | 67.6 |
|  |  | ART singleton | 5270 | 0.23 |
|  |  | NC multiples | 19 803 | 0.87 |
|  |  | ART multiples | 903 | 0.04 |
|  |  | No continuation | 713 941 | 31.3 |
|  |  | Total | 2 284 700 | 100 |
| **NC****Perinatal loss** |  | NC singleton | 11 101 | 80.5 |
|  |  | ART singleton | 213 | 1.5 |
|  |  | NC multiples | 196 | 1.4 |
|  |  | ART multiples | 35 | 0.25 |
|  |  | No continuation | 2242 | 16.3 |
|  |  | Total | 13 787 | 100 |
| **ART****Surviving child** |  | NC singleton | 15 486 | 27.2 |
|  |  | ART singleton | 11 793 | 20.7 |
|  |  | NC multiples | 527 | 0.93 |
|  |  | ART multiples | 2003 | 3.5 |
|  |  | No continuation | 27 101 | 47.6 |
|  |  | Total | 56 910 | 100 |
| **ART****Perinatal loss** |  | NC singleton | 100 | 21.8 |
|  |  | ART singleton | 166 | 36.2 |
|  |  | NC multiples | 8 | 1.7 |
|  |  | ART multiples | 39 | 8.5 |
|  |  | No continuation | 145 | 31.7 |
|  |  | Total | 458 | 100 |
| Arrows and colours refer to the legend of Figure 2, to help illustrate how the selection into the discordant sibships occur. Abbreviations: ART – assisted reproductive technology, NC- natural conception. | | | | |

| **Table S4.** **Characteristics of 1^st^ and 2^nd^ consecutive singleton deliveries with and without perinatal death of the firstborn singleton, categorized by conception method of 1^st^ and 2^nd^ singleton** | | | | | | | | | | | | | | | | | |
| --- | --- | --- | --- | --- | --- | --- | --- | --- | --- | --- | --- | --- | --- | --- | --- | --- | --- |
| **Characteristic** | **Outcome firstborn singleton** | **Combinations of conception methods in consecutive singleton deliveries** | | | | | | | | | | | | | | | |
|  |  | **Sibship NC-NC**  **n = 1 963 564** | | | | **Sibship NC-ART**  **n = 12 653** | | | | **Sibship ART-NC**  **n = 20 750** | | | | **Sibship ART-ART**  **n = 16 187** | | | |
| Mean years trying to conceive before 1^st^ pregnancy (SD) ^a^ | Surviving firstborn | 2.3 (1.8) | | | | 2.8 (2.0) | | | | 3.6 (2.0) | | | | 3.5 (2.0) | | | |
|  | Perinatal death of firstborn | 2.6 (2.1) | | | | 3.3 (2.6) | | | | 3.8 (1.6) | | | | 3.7 (2.0) | | | |
| Mean years between 1^st^ and 2^nd^ birth (SD) | Surviving firstborn | 3.2 (1.9) | | | | 5.8 (3.1) | | | | 2.6 (1.4) | | | | 3.1 (1.3) | | | |
|  | Perinatal death of firstborn | 2.4 (2.0) | | | | 3.8 (2.9) | | | | 2.4 (1.6) | | | | 2.1 (1.2) | | | |
|  |  | **1^st^ birth NC** | | **2^nd^ birth NC** | | **1^st^ birth NC** | | **2^nd^ birth ART** | | **1^st^ birth ART** | | **2^nd^ birth NC** | | **1^st^ birth ART** | | **2^nd^ birth ART** | |
|  |  | **n** | **%** | **n** | **%** | **n** | **%** | **n** | **%** | **n** | **%** | **n** | **%** | **n** | **%** | **n** | **%** |
| Caesarian section | Surviving firstborn | 291 829 | 15.0 | 254 541 | 13.1 | 2725 | 22.1 | 2990 | 24.2 | 4950 | 24.0 | 4186 | 20.3 | 3548 | 22.2 | 3456 | 21.7 |
|  | Perinatal death of firstborn | 2545 | 19.4 | 4231 | 32.3 | 67 | 21.9 | 136 | 44.4 | 33 | 26.4 | 57 | 45.6 | 52 | 23.0 | 104 | 46.0 |
| Induction of labour | Surviving firstborn | 249 771 | 12.8 | 217 249 | 11.1 | 1750 | 14.2 | 2068 | 16.8 | 3900 | 18.9 | 2890 | 14.0 | 2954 | 18.5 | 2597 | 16.3 |
|  | Perinatal death of firstborn | 5532 | 42.2 | 4120 | 31.4 | 140 | 45.8 | 109 | 35.6 | 54 | 43.2 | 45 | 36.0 | 110 | 48.7 | 89 | 39.4 |
| Preterm birth,  <37 weeks | Surviving firstborn | 101 357 | 5.2 | 69 700 | 3.6 | 801 | 6.5 | 832 | 6.7 | 1377 | 6.7 | 924 | 4.5 | 1050 | 6.6 | 883 | 5.5 |
|  | Perinatal death of firstborn | 6686 | 51.0 | 1663 | 12.7 | 168 | 54.9 | 60 | 19.6 | 69 | 55.2 | 20 | 16.0 | 143 | 63.3 | 37 | 16.4 |
| Very preterm birth, <32weeks | Surviving firstborn | 10 228 | 0.52 | 9150 | 0.47 | 101 | 0.82 | 123 | 1.0 | 187 | 0.91 | 128 | 0.62 | 153 | 0.96 | 144 | 0.90 |
|  | Perinatal death of firstborn | 4014 | 30.6 | 390 | 3.0 | 108 | 35.3 | 19 | 6.2 | 46 | 36.8 | 7 | 5.6 | 105 | 46.5 | 7 | 3.1 |
| Abbreviations: ART – assisted reproductive technology, NC – natural conception, SD – standard deviation. ^a^ Sub-population from Sweden (n=118 707) with known time trying to conceive (surviving firstborn: NC-NC n=56 268, NC-ART n=1256, ART-NC n=9180, ART-ART n=7312, perinatal death of firstborn: NC-NC n=386, NC-ART n=53, ART-NC n=44, ART-ART n=73) | | | | | | | | | | | | | | | | | |
